# Identification of a bone morphogenetic protein type 2 receptor neutralizing antibody

**DOI:** 10.1186/s13104-019-4367-0

**Published:** 2019-06-11

**Authors:** Ruthann E. Gorrell, Madeline H. Totten, Laura J. Schoerning, Jordan B. Newby, Logan J. Geyman, Warren G. Lawless, Julia M. Hum, Jonathan W. Lowery

**Affiliations:** Division of Biomedical Science, Marian University College of Osteopathic Medicine, 3200 Cold Spring Rd, Indianapolis, IN 46222 USA

**Keywords:** Bone morphogenetic protein, Bone morphogenetic protein receptor type 2, Neutralizing antibody, BMP, BMPR2, SMAD

## Abstract

**Objective:**

The bone morphogenetic protein (BMP) signaling pathway comprises the largest subdivision of the transforming growth factor (TGFβ) superfamily. BMP signaling plays essential roles in both embryonic development and postnatal tissue homeostasis. Dysregulated BMP signaling underlies human pathologies ranging from pulmonary arterial hypertension to heterotopic ossification. Thus, understanding the basic mechanisms and regulation of BMP signaling may yield translational opportunities. Unfortunately, limited tools are available to evaluate this pathway, and genetic approaches are frequently confounded by developmental requirements or ability of pathway components to compensate for one another. Specific inhibitors for type 2 receptors are poorly represented. Thus, we sought to identify and validate an antibody that neutralizes the ligand-binding function of BMP receptor type 2 (BMPR2) extracellular domain (ECD).

**Results:**

Using a modified, cell-free immunoprecipitation assay, we examined the neutralizing ability of the mouse monoclonal antibody 3F6 and found a dose-dependent inhibition of BMPR2-ECD ligand-binding. Consistent with this, 3F6 blocks endogenous BMPR2 function in the BMP-responsive cell line HEK293T. The specificity of 3F6 action was confirmed by demonstrating that this antibody has no effect on BMP-responsiveness in HEK293T cells in which *BMPR2* expression is knocked-down. Our results provide important proof-of-concept data for future studies interrogating BMPR2 function.

## Introduction

The TGF-β superfamily is a group of pleiotropic cytokines and their receptors that contribute to metazoan cellular development and regulation [1]. Extracellular dimeric ligands bind to transmembrane serine/threonine kinase receptor complexes, bringing together two type 1 and two type 2 receptors, in order to activate a group of effectors called SMAD proteins [1–3]. The largest subdivision of this superfamily is comprised of the bone morphogenetic proteins (BMPs), which activate SMADs 1, 5, and 8 and play an essential role in embryonic development and in postnatal tissue homeostasis [4]. Moreover, dysregulated BMP signaling underlies numerous human pathologies ranging from pulmonary arterial hypertension to heterotopic ossification [5]. Thus, understanding of the basic mechanisms by which BMP signaling occurs and is regulated is a highly important goal and may yield translational opportunities.

Functional studies evaluating BMP pathway mechanics, such as global or conditional genetic knockout of specific components, have been complicated by developmental requirements and/or the ability of pathway components to compensate for one another [4]. Additionally, the translational potential of these strategies is questionable due to ethical concerns and technical limitations [5]. This has led to the development of several pharmacologically-based strategies, including decoy receptors and small molecule inhibitors, for non-genetic based modulation of BMP pathway activity [6]. That said, the repertoire of these molecules remains limited and, in particular, few tools exist for inhibiting the function of type 2 BMP receptors, the activity of which are essential for initiating signal transduction.

In this study, we sought to identify and validate a commercially available antibody that neutralizes the ligand-binding function of bone morphogenetic protein receptor type 2 (BMPR2), which is essential for embryogenesis and has been shown to play clinically-relevant roles in pulmonary vascular homeostasis and remodeling of the postnatal skeleton [5]. We developed a modified, cell-free immunoprecipitation assay quantified by ELISA and found that the mouse monoclonal antibody 3F6 inhibits ligand-binding by BMPR2 in a dose-dependent manner. Additionally, using a BMP-responsive cell line we found that pre-treatment with 3F6 leads to reduced sensitivity in response to BMP pathway activation by BMP2. These results provide important proof-of-concept data for future studies interrogating BMPR2 function in numerous physiological and pathophysiological contexts.

## Main text

### Materials and methods

#### Modified immunoprecipitation assay and ELISA

Ligand-binding by BMPR2-ECD was performed as previously described [7] with the modifications detailed below. BMPR2-ECD/Fc fusion (Sino Biologicals 10551-H03H) was mixed with 5 µL Protein G-coupled Dynabeads (Invitrogen 1003D) at room temperature for 30 min in 200 µL total volume with gentle rocking. The loaded beads were then washed twice with PBS and resuspended in 135 μL PBS ± 3F6 (Thermo Fisher Scientific Cat# MA5-15827) or control ascites (Sigma M8273) and mixed for 1 h at room temperature with gentle rocking. 800 ng recombinant human BMP2 (R&D Systems 892143) was added to the beads and incubated overnight at 4 °C with gentle rocking. The supernatant was removed and examined using a Quantikine Human BMP2 Immunoassay ELISA (R&D Systems DBP200) according to the manufacturer’s instructions.

#### Cell-based BMP2-responsiveness assay

HEK293T cells, obtained from ATCC, were cultured in DMEM supplemented with 10% fetal bovine serum (FBS) (Gibco), hereafter referred to as “10% DMEM,” and grown at 37 °C in 5% CO_2_. Cells were not tested for mycoplasma contamination. For BMP2-responsiveness assays, cells were passaged at 1 million per well in 6-well dishes in 10% DMEM on day 0; on day 1 the medium was exchanged to DMEM containing 0.5% FBS. After approximately 24 h of serum restriction, select wells were treated with 250 ng/mL 3F6 or control ascites for 30 min followed by 100 ng/mL rhBMP2 for 4 h.

##### Knockdown of BMPR2 expression

1.6 × 10^4^ cells in 100 µL 10% DMEM were added to wells of a 96-well plate. The cells were incubated overnight at 37 °C in a humidified incubator in an atmosphere of 5% CO_2_. After 18–20 h, the media was aspirated from the wells. Next, 110 µL of 10% DMEM containing 8 μg/mL hexadimethrine bromide was added to each well and the plate was gently swirled to mix. To each well, lentivirus containing scramble control (Sigma SHC002V) or anti-human BMPR2 shRNA (Sigma TRCN0000000460) were added and the plate was gently swirled to mix. The volume of virus added was calculated using a multiplicity of infection of 5. The cells were incubated overnight at 37 °C in a humidified incubator in an atmosphere of 5% CO_2_. After 18–20 h, the media containing lentiviral particles was removed from the wells. A volume of 120 μL of fresh 10% DMEM was added to each well, and the plate was returned to the incubator. After 18–20 h, the media was removed from the wells, and fresh media containing 2 µg/mL puromycin in 10% DMEM was added in order to select for the transduced cells. The media was replaced with fresh puromycin-containing media every 3–4 days. When the cells reached 80% confluence, they were expanded, continuing to be maintained in puromycin-containing media. RNA was collected from scramble control and *BMPR2* knock-down (*BMPR2*-KD) HEK293T cells using the RNEasy Plus Universal Kit (QIAGEN). cDNA was generated using SuperScript III First-Strand Synthesis Kit (Thermo Fisher Scientific 18080051). Quantitative RT-PCR was performed using TaqMan probes targeting *BMPR2* (Thermo Fisher Scientific Hs00176148) and *HPRT1* (Thermo Fisher Scientific Hs99999909); data were analyzed using the 2^−∆∆Ct^ method and normalized to scramble control. Immunblots to confirm reduced BMPR2 protein level were described as below.

#### Immunoblots

Immunoblots were performed on protein isolates from HEK293T cells after lysis in RIPA buffer (50 mM Tris Base, 150 mM NaCl, 1% NP-40, 0.5% sodium deoxycholate, 0.1% SDS, pH 8.0) supplemented with Halt Protease and Phosphatase Inhibitor Cocktail (Thermo). Lysates were resolved by SDS-PAGE and transferred to Amersham Hybond ECL nitrocellulose membranes (GE Healthcare). All samples were denatured by heating at 100 °C for 10 min after mixing with 6× reducing sample buffer (60% glycerol, 300 mM Tris pH 6.8, 12 mM EDTA, 12% SDS, 864 mM 2-mercaptoethanol, 0.05% bromophenol blue). After blocking in 10% milk in PBST (PBS + 0.1% Tween-20), the following primary antibodies (1:250) were applied in 5% milk in PBST: anti-BMPR2 C-terminal domain (BD Biosciences, 612292), anti-phosphorylated SMAD1, 5, and 8 (Cell Signaling 9516 and 13820), anti-SMAD1 (Cell Signaling 6944), and anti-β-actin (Sigma A2228). Appropriate HRP-conjugated species-specific goat polyclonal secondary antibodies (1:1000; anti-mouse: Kirkegaard & Perry Laboratories, 04-18-06; and anti-rabbit: Cell Signaling, 7074) were utilized and western blots were developed by chemiluminescence using WesternBright Quantum or Sirius substrate (Advansta). Stripping of membranes for re-probing was accomplished using Gentle Review Stripping Buffer (VWR). Western blots were visualized using a LiCor C-Digit imager and quantified by ImageJ (ImageJ, RRID:SCR_003070).

#### Statistical analyses

Statistical analyses were performed using GraphPad Prism 5 as described in each respective figure legend or in the text. A p-value of < 0.05 was considered significant.

### Results

#### Assay development

We first established a modified immunoprecipitation assay wherein recombinant BMP2 was pulled down by BMPR2-ECD conjugated to Protein G beads; the unbound BMP2, found in the supernatant, was subsequently quantified by ELISA. A pilot dose–response series (data not shown) using beads loaded with 0.5 µg to 3.0 µg BMPR2-ECD while holding BMP2 concentration constant led us to further optimize the assay using 2 µg BMPR2-ECD; this led to a 73% reduction in BMP2 signal (mean ± SEM: 73.00 ± 7.077; p < 0.0001 by paired t-test, n = 11), thus confirming the ligand-binding activity of BMPR2-ECD in this assay.

#### Identification of a putative neutralizing antibody

**Fig. 1:**
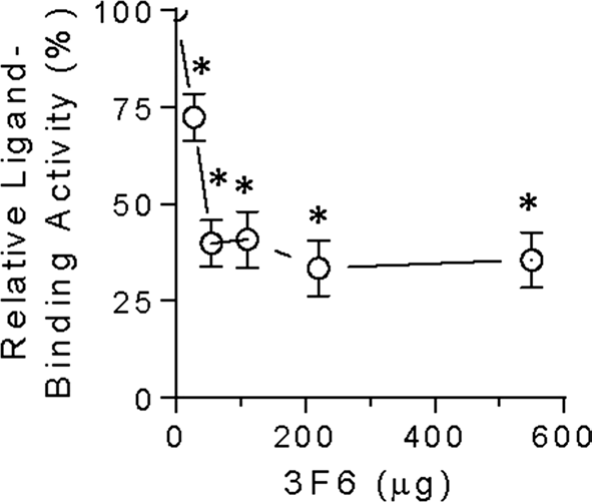
Antibody 3F6 reduces BMPR2-ECD ligand-binding activity in a modified immunoprecipitation assay. Neutralizing the ligand-binding activity of BMPR2-ECD using various amounts of 3F6. Results are quantified by ELISA and expressed as mean ± SEM relative to the ligand-binding activity of BMPR2-ECD in the absence of 3F6. n ≥ 3 per condition. Asterisk indicates p < 0.05 by paired t test

We then sought to identify an antibody capable of neutralizing the ligand-binding activity of the BMPR2-ECD. This led us to examine 3F6, which is a mouse monoclonal antibody raised against the N-terminus of BMPR2, and found a dose-dependent inhibition of BMPR2-ECD ligand-binding (Fig. 1); in this experimental design, the inhibition appears to saturate at an approximate ratio of 2 µg BMPR2-ECD: 25 µg 3F6. Given that the commercial availability of this antibody is as an ascites preparation, specificity of this assay was confirmed by demonstrating that ligand-binding activity of BMPR2-ECD is unchanged in the presence of non-specific, negative control ascites (p = 0.9135 by paired t test, n = 3).

#### Validation of neutralizing activity in a cell-based assay

**Fig. 2:**
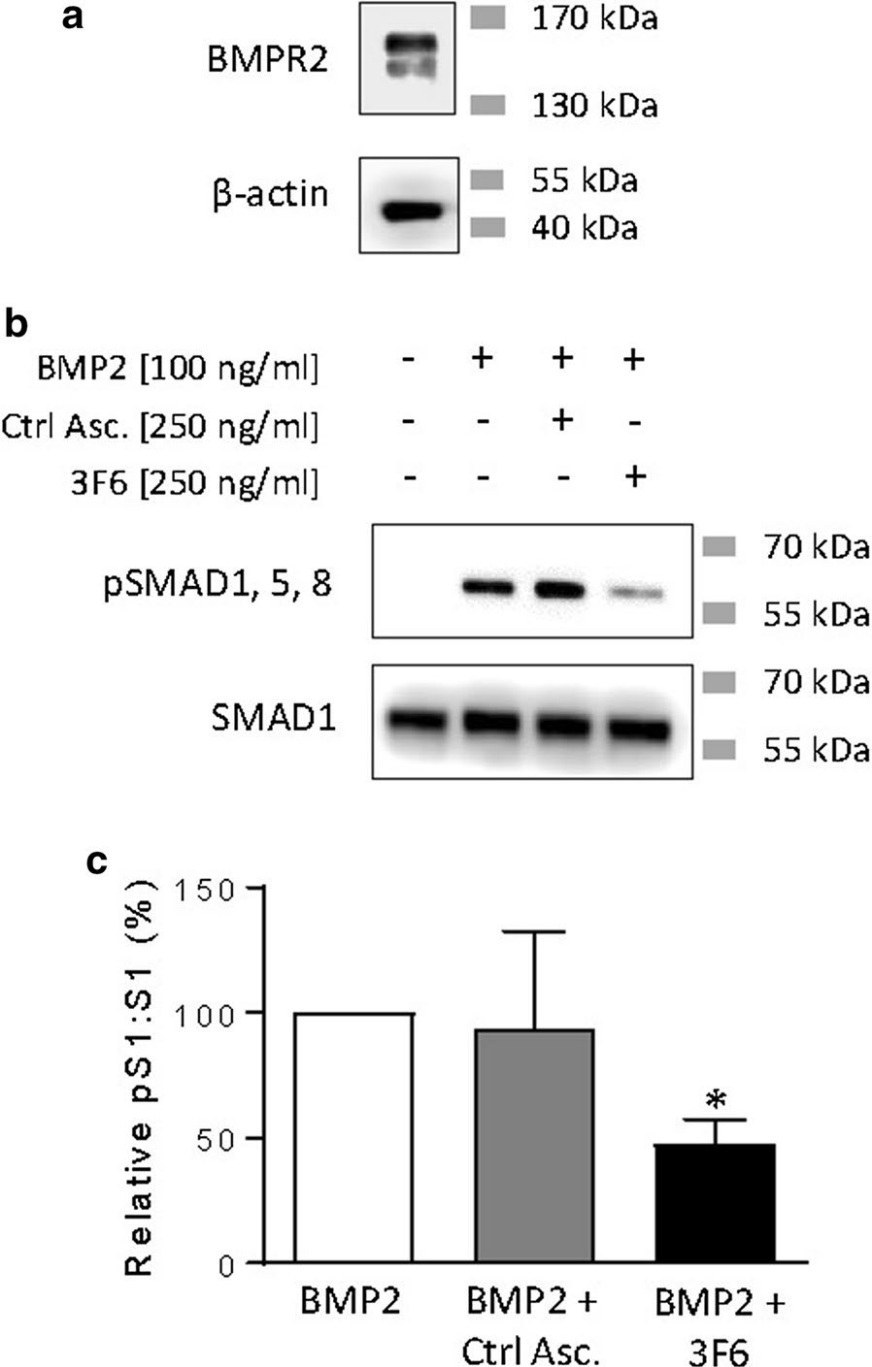
Antibody 3F6 reduces activation of BMP pathway in HEK293T cells. **a** Expression of endogenous BMPR2 by HEK293T cells compared to β-actin loading control. Approximate molecular weights are indicated. **b**, **c** BMP2 induces phosphorylation of SMAD1, 5, and 8 (pSMAD1,5,8) in HEK293T cells and this response is blunted by pre-treatment with 3F6. Approximate molecular weights are indicated in **b**. Results are quantified in **c** and expressed as mean ± SEM ratio of phosphorylated SMAD1,5,8: total SMAD1 relative to BMP2 treatment alone. No effect on BMP2-responsiveness was observed with pre-treatment using control ascites (Ctrl Asc.). n = 3 per condition. Asterisk indicates p < 0.05 by paired t test

We next established a cell-based assay to test the hypothesis that 3F6 pretreatment attenuates the BMP-responsiveness of HEK293T cells, which express BMPR2 endogenously (Fig. 2a) and mount a robust activation of SMAD1, 5, and 8 in response to exogenous BMP2 (Fig. 2b). Pre-treatment with control ascites had no effect on the BMP2-induced pathway activation (Fig. 2b, c), but the 3F6 antibody did in fact blunt the cellular response to BMP2 (Fig. 2b, c).

**Fig. 3:**
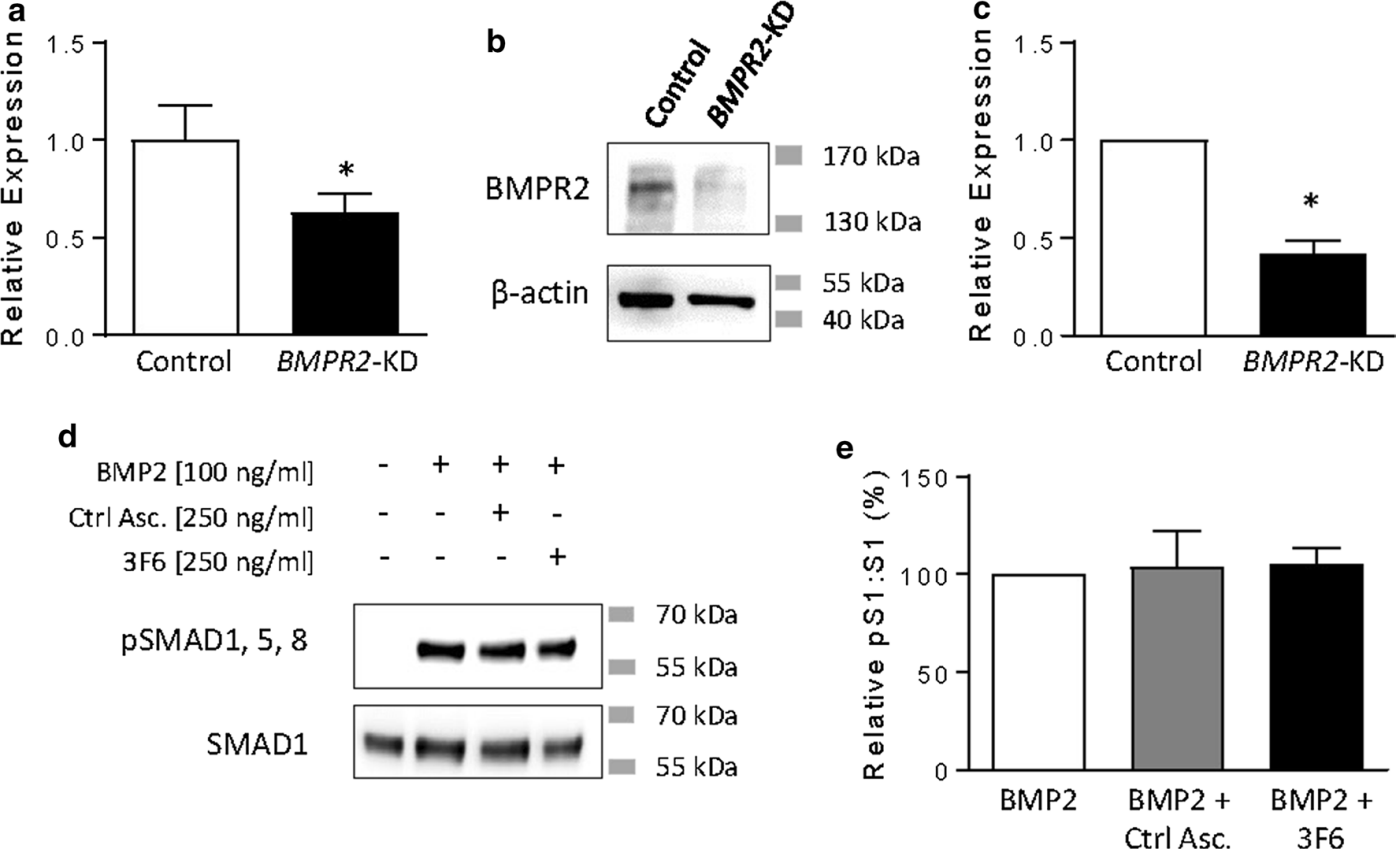
Antibody 3F6 has no effect on BMP-responsiveness in *BMPR2* knock-down HEK293T cells. **a** Expression levels of endogenous *BMPR2* in scramble control HEK293T cells (Control) and HEK293T cells carrying anti-*BMPR2* shRNA (*BMPR2*-KD) compared to *HPRT1* loading control. Data are expressed as normalized to scramble control (Control) using the 2^−∆∆Ct^ method. n = 6 per condition. Asterisk indicates p < 0.05 by unpaired t test. **b**, **c** Expression of endogenous BMPR2 in scramble control HEK293T cells (Control) and *BMPR2*-KD HEK293T cells compared to β-actin loading control. Approximate molecular weights are indicated in **b**. Representative immunoblot is shown in **b** and results from three independent runs are quantified in **c** (data are expressed as mean ± SEM ratio of BMPR2: β-actin normalized to scramble control (Relative Expression)). Asterisk indicates p < 0.05 by paired t test. **d**, **e** BMP2 induces phosphorylation of SMAD1, 5, and 8 (pSMAD1,5,8) in *BMPR2*-*KD* HEK293T cells. No effect on BMP2-responsiveness of *BMPR2*-*KD* HEK293T cells was observed with pre-treatment using control ascites (Ctrl Asc.) or 3F6. Approximate molecular weights are indicated in **d**. Results are quantified in E and expressed as mean ± SEM ratio of phosphorylated SMAD1,5,8: total SMAD1 relative to BMP2 treatment alone (Relative pS1:S1). n = 4 per condition

To confirm the specificity of 3F6 against BMPR2, we used lentiviral transduction to generate HEK293T cells in which *BMPR2* expression is stably knocked-down by shRNA; reduced *BMPR2* transcript and BMPR2 protein levels were confirmed by quantitative RT-PCR (Fig. 3a) and immunoblot (Fig. 3b, c), respectively. 3F6 had no impact on the BMP2-responsiveness in this cell line (Fig. 3d, e).

### Discussion

The BMP signaling pathway plays essential roles in normal metazoan development and tissue homeostasis [4]. Remarkably, the same basic pathway architecture is conserved throughout evolution: extracellular dimeric ligands interact with complexes of type 1 and type 2 receptors on the cell surface to activate a class of intracellular effectors and regulate cellular differentiation and/or behavior via genomic and non-genomic means. Gene duplication events are thought to have given rise to the highly homologous, complex, and promiscuous BMP pathway present in mammals. This complexity creates significant challenges for mechanistic studies examining the BMP pathway in model organisms and, moreover, devising strategies for modulating the activity of specific BMP pathway components to advance human health. This has led some investigators to develop monoclonal antibodies that neutralize the activity of BMP pathway components; notable examples include neutralizing antibodies against the extracellular antagonists Noggin and Gremlin and the integral transmembrane receptors ACVR2A, ACVR2B, and ACVR1 [8–13].

In this study, we sought to identify antibody(ies) capable of blocking the activity of BMPR2. We started our search with three strict criteria: (1) the antibody must be raised against the region of BMPR2 exposed to the extracellular environment, i.e., the N-terminal ligand-binding domain; (2) the antibody must be monoclonal to promote specificity of action; and (3) the antibody must be purchasable from a commercial source so that it may be readily available to the field. 3F6 from Thermo Fisher Scientific met these qualifications. No funding of any kind was sought from or received from this vendor. Using a cell-free immunoprecipitation assay quantified by ELISA, we determined that 3F6 is capable of blocking ligand-binding by recombinant BMPR2-ECD. We then extended this work to examine neutralization of endogenous BMPR2 by 3F6 in HEK293T cells and obtained consistent results.

In the course of this study, we also performed preliminary analyses on 9A10 and 1F12, both of which are mouse monoclonal antibodies raised against the N-terminus of BMPR2 (produced by Abcam and Thermo Fisher Scientific, respectively). We were unable to achieve neutralizing ability by 9A10 in our cell-free assay and this antibody was eliminated from further analysis. In contrast, pilot data suggest that 1F12 is capable of blocking ligand-binding by recombinant BMPR2-ECD; we were unable to further validate this result in our cell-based assay due to challenges with consistent availability from the vendor.

Given that BMPR2 is widely expressed, our results provide proof-of-concept data for a novel strategy whereby investigators may inhibit BMPR2 function in various physiological contexts. When coupled with other inhibitors of the BMP pathway [6], 3F6 may provide novel insights into the mechanisms by which BMP signaling regulates embryogenesis and postnatal tissue homeostasis.

## Limitations

The primary limitation of this study is the fact that the characterization and validation of 3F6 was performed in HEK293T cells as opposed to primary cells or in vivo. This approach was advantageous due to the ease of use, availability, ability to be stably transduced by lentivirus, and strong BMP-responsiveness of these cells. Additionally, our study is limited by the examination of 3F6’s ability to block interaction of BMPR2 with a single ligand, namely BMP2. Structural studies indicate that all BMP ligands interact with the same region on the extracellular face of BMPR2 [14] and that the affinity of BMPR2 for BMP2 is relatively similar to that of other ligands [15]. That said, given these limitations, we encourage investigators to examine the functional impact and specificity of 3F6 treatment on primary cells and/or tissues in their own context(s) of interest.

## Data Availability

The datasets used and/or analysed during the current study are available from the corresponding author on reasonable request.

## Abbreviations

ACVR2A: activin A receptor type 2A
ACVR2B: activin A receptor type 2B
ACVR1: activin A receptor type 1
BMP: bone morphogenetic protein
BMPR2: BMP type 2 receptor
BMPR2-ECD: BMPR2 extracellular domain
ELISA: enzyme-linked immunosorbent assay
HEK293T: human embryonic kidney 293 transformed
TGFβ: transforming growth factor-β
